# Facing the partner influences exchanges in force

**DOI:** 10.1038/srep35397

**Published:** 2016-10-14

**Authors:** Atsushi Takagi, Carlo Bagnato, Etienne Burdet

**Affiliations:** 1 Department of Bioengineering, Imperial College London, South Kensington Campus, London SW7 2AZ, UK

## Abstract

Many studies in psychology have documented how the behaviour of verbally communicating pairs is affected by social factors such as the partner’s gaze. However, few studies have examined whether physically interacting pairs are influenced by social factors. Here, we asked two partners to exchange forces with one another, where the goal was to accurately replicate the force back onto the other. We first measured an individual’s accuracy in reproducing a force from a robot. We then tested pairs who knowingly exchanged forces whilst separated by a curtain. These separated pairs exchanged forces as two independent individuals would, hence the force reproduction accuracy of partners is not affected by knowingly reproducing a force onto a nonvisible partner. On the other hand, pairs who exchanged forces whilst facing one another consistently under-reproduced the partner’s force in comparison to separated partners. Thus, the force reproduction accuracy of subjects is strongly biased by facing a partner.

The literature in psychology has documented social factors that affect the attitudes of verbally interacting partners^1^,^2^,^3^,^4^,^5^. The degree to which a listener likes a communicator, determined using a questionnaire after interaction, is influenced by several factors such as the physical distance between the two and the communicator’s posture and orientation of their body^2^. Gaze in particular has a powerful effect on human behavioural responses. The ability to process and follow another’s gaze is also critical to social development^6^,^7^, and a study found that the distance one maintains when approaching a human or virtual avatar depends on whether the approached is looking at you^5^, and that people maintain a larger distance when approaching from the front rather than the back of an avatar^8^. Another study found that, a target presented in the same direction of the gaze of a face presented on screen is detected faster than when the target appears opposite to the gaze direction^9^. In another study, when subjects had to determine the orientation of the head, their initial gaze still landed on the eyes roughly 50% of the time^10^. Although these studies^11^,^12^ show a significant influence of gaze on verbal interaction and social behaviour, relatively little is known whether physical, sensorimotor interaction between partners can be influenced by social factors, such as gaze, involved in facing a partner.

Physical interaction occurs when a parent coordinates their movements to help a child learn to walk, and when a therapist supports a patient to recover motor functions after injury or disease. It is still unclear how physically interacting partners modify their behaviour to enable such joint actions^13^ and there is little understanding in literature of whether social factors influence joint action. As a result of this lack of knowledge, it is difficult to identify whether an interactive strategy observed between pairs during sensorimotor interaction is the outcome of social influences or stems from motor adaptation. To enrich our understanding of the influence of social factors involved in facing a partner on sensorimotor interaction, we asked pairs to exchange forces, where the goal was to reproduce the partner’s force accurately. We tested two configurations, one where partners were separated by a curtain and another where partners faced one another. This would help us test whether social factors involved in facing a partner, such as gaze, affect the ability to sense and reproduce a partner’s force.

## Results

**Figure 1:**
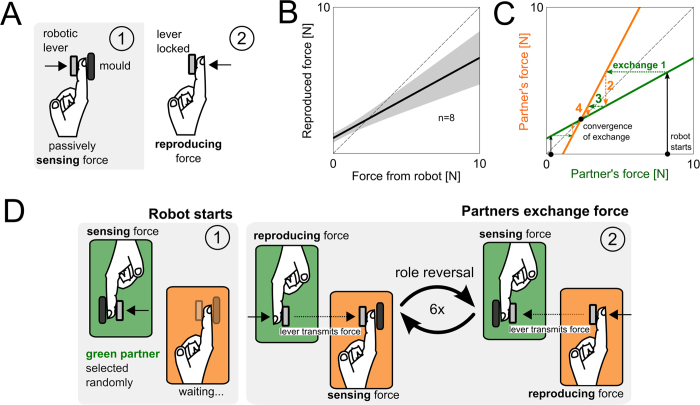
The prediction of paired force exchanges if they behave like two individuals, and the experimental protocol of exchanging forces. (**A**) Top-down view of individuals reproducing a force from a robot. The finger is sandwiched between a robotic lever and a mould to passively sense the force. During reproduction, the lever is locked and the same finger is used to reproduce the force against it. (**B**) Reproduced force as a function of force from the robot for individuals, who over-reproduced forces smaller, and under-reproduced ones larger than 2N (shaded region is the 95% confidence interval of a linear mixed-effects model fit). (**C**) Prediction of force exchanges between pairs if they behave like individuals. The convergence of the exchange should be at the intersection between the individual (green/orange traces) and perfect (dashed black trace) reproduction curves. (**D**) Experimental protocol for pairs exchanging forces. Each partner had their own robotic lever to sense and reproduce forces. The levers were rigidly connected through software to enable partners to exchange forces. (1) The robot selects a random partner who reproduces this force onto the other subject. (2) The force is exchanged 6 times, after which the robot reinitiates the exchange with another force picked from 2, 4, 6, 8 or 10N.

To determine whether partners exchanging forces are influenced by facing a partner, we first measured the accuracy of individuals in reproducing a force from a robot. Eight right-handed subjects (4 male, 4 female) were asked to passively sense a force applied on the right index finger, which was squeezed between a robotic lever and a mould supporting the finger (see Fig. 1A); after sensing, individuals reproduced the perceived force back onto the lever using the same finger. The robot sent 30 forces, which were picked randomly from a uniform distribution between 0.5–10N. No feedback concerning the accuracy of the reproduced force was given to subjects. The results from this individual force reproduction experiment revealed that individuals over-reproduced forces smaller than 2N and under-reproduced larger forces (see Fig. 1B). Based on this control, we can predict the behaviour of two individuals exchanging forces with one another. A pair of individuals would converge to a force corresponding to the intersection between the individual force reproduction and the perfect reproduction curves (see Fig. 1C). Thus, both small and large forces should converge after a few exchanges to the intersection of these curves. Do pairs exchange forces like two individuals would?

### Force exchanges between separated partners

**Figure 2:**
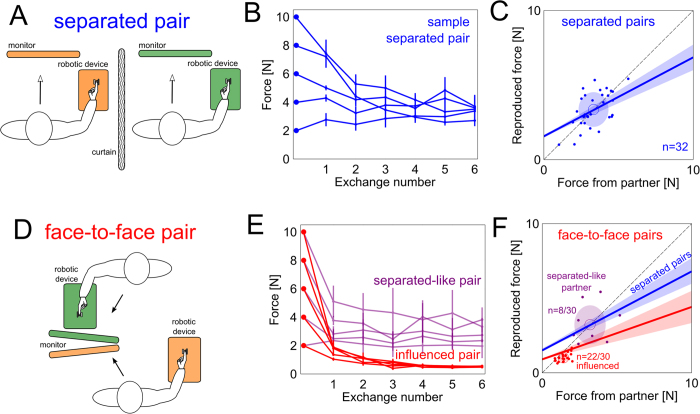
Separated pairs behaved differently to most face-to-face pairs who were influenced by the configuration of the experimental setup. (**A**) Configuration of the experimental setup for separated pairs who exchanged forces separated by a curtain. Separated pairs were aware of the fact that they exchanged forces with their partner. (**B**) Exchanged force as a function of the exchanges for a sample separated pair (standard errors shown). (**C**) Reproduced force as a function of force from the partner for separated pairs. Each point corresponds to a single partner’s mean force from partner plotted against their mean reproduced force. The blue ellipsoid is the mean and standard error of the convergence of the force exchanges. Separated pairs converge to a force approximately where two individuals are expected to converge in Fig. 1C. (**D**) Face-to-face experimental configuration, where the same protocol (see Fig. 1D) as the separated pair was employed. If pairs are not influenced by the facing configuration, face-to-face pairs should converge to force exchanges similar to that observed with separated pairs. (**E**) Forces exchanged from separated-like and influenced sample face-to-face pairs. Separated-like pairs exchanged forces like separated pairs, but influenced pairs consistently under-reproduced the partner’s force and the convergence of exchanges was significantly lower. (**F**) Force reproduction of face-to-face pairs (red trace) was significantly different to separated pairs (blue trace). Furthermore, two prominent behaviours were observed where partners either behaved similarly to those from separated pairs (violet ellipse, n = 8/30) or were influenced by the face-to-face configuration (red ellipse, n = 22/30). The convergence of the exchange for the subpopulation who were influenced was significantly lower than what should be expected from separated pairs.

We recruited 16 pairs (8 male-male, 8 female-female, all right-handed) who were asked to exchange the same force with one another. Importantly, these pairs were separated by a curtain and sat side-by-side (see Fig. 2A) but were aware that they would exchange forces with a partner. Each partner in a pair had their own robotic lever, and the two levers were rigidly connected, through software, during exchanges. The experiment was composed of 15 trials where each trial was initiated by a robot that picked, in random order, a force level of 2, 4, 6, 8 or 10N which was applied to the finger of a randomly selected partner (see Fig. 1D). This force was exchanged 6 times between the pair at every trial. Subjects were instructed to “reproduce the same force they had just perceived” and received all other instructions from a computer monitor. Verbal communication was prohibited throughout the exchanges, and pairs were strangers to one another.

Figure 2B shows all exchanges from a sample separated pair that converged to exchanging forces between 2–3N. This behaviour is what we expect if two individuals exchange forces. To visualise the force to which separated pairs converged, the mean force reproduced by each partner is plotted as a function of the mean force from their partner (blue points on Fig. 2C). The convergence of the force exchanges corresponds to the prediction of two individuals who exchange forces. Thus, the behaviour of separated pairs is what is expected of two individuals who sense and reproduce forces independently.

### Force exchanges between face-to-face partners

If social factors do not influence pairs who exchange forces with one another, then pairs who face one another (see Fig. 2D) should exchange forces like separated pairs, and should reproduce a partner’s force like individuals reproducing a robot’s force. We asked another 15 pairs (8 male-male, 7 female-female, all right-handed) to exchange forces as separated pairs did, but whilst seated face-to-face opposite one another (see Fig. 2D). Figure 2E shows all the force exchanges from two sample face-to-face pairs; some pairs exchanged forces as separated pairs did (violet traces), but most pairs under-reproduced the partner’s force (red traces) such that the convergence of the exchange was much lower than in separated pairs. The red trace in Fig. 2F shows the reproduced force of face-to-face partners as a function of the force from the partner. Linear mixed-effects analysis revealed a significant effect of the face-to-face configuration on the force reproduction curve compared with separated partners (χ^2^(2) = 16.5, p < 0.0003). Furthermore, the mean exchanged force of the face-to-face partners violated normality (Shapiro-Wilk test, p < 10^−3^), which is indicative of a subpopulation of face-to-face pairs exchanging different levels of force in comparison to others. We fit a Gaussian mixture model on the data from face-to-face pairs only and found two prominent subpopulations of face-to-face partners (violet and red ellipses in Fig. 2F). One-way analysis of variance of the mean exchanged force with subpopulation as a factor revealed that at least one of the subpopulations of face-to-face partners was significantly different from the mean force exchanged by separated partners (F(2,61) = 49.88, η^2^ = 0.63, p < 10^−12^); one subpopulation of 8/30 face-to-face partners (violet ellipse, Fig. 2F) had a mean exchanged force similar to separated partners (post-hoc Tukey test, p > 0.997), and another larger subpopulation of 22/30 partners (red ellipse, Fig. 2F) were influenced by the face-to-face configuration and exchanged a different mean force to separated partners (p < 10^−9^). These influenced face-to-face partners greatly under-reproduced the partner’s force, and so the exchanged force rapidly decayed. Furthermore, all influenced face-to-face partners were paired with one another, i.e. all face-to-face partners within a pair were either both influenced or neither were. This demonstrates a strong coupling effect when pairs face one another, such that one partner under-reproducing the force causes the other partner to also under-reproduce the force. Hence, both face-to-face partners must be resilient to the facing configuration to reproduce forces as separated partners do. Thus, facing the partner, and the coupling effect associated with one partner under-reproducing the force, significantly influences a subject’s accuracy in reproducing a partner’s force.

## Discussion

We first assessed the accuracy of individuals when reproducing forces from a robot, which was used to predict roughly what to expect when two individuals independently exchange forces with one another. We then tested pairs, whose goal was to exchange the same force with one another, in two configurations. Pairs separated by a curtain reproduced the partner’s force as expected of two individuals exchanging forces. Thus, the knowledge of exchanging forces with another partner does not change the accuracy in reproducing a partner’s force. However, a majority of partners who exchanged forces whilst facing each other were strongly influenced and under-reproduced the partner’s force in comparison to separated partners. Thus, facing a partner reduces the accuracy in reproducing a partner’s force.

Our study is related to a previous study^14^ by Shergill *et al*., who found that pairs exchanging a force escalated to a force magnitude similar to what we have observed. However, subjects were not tested in higher force ranges, in contrast to our study. If the escalation in force is explained by how individuals over-reproduce an external force, as suggested by Shergill *et al*., then an indefinite escalation in the exchanged force is expected. However, our data show that paired force exchanges converge. This discrepancy between our results of converging force and Shergill *et al*.’s prediction of indefinite escalation can be explained by the way in which individual force reproduction was tested by Shergill *et al*. In their study, individuals were asked to passively sense a force on the index finger, and then to reproduce this force back onto the same finger using their contralateral finger; this two-finger reproduction is different from how pairs experience the task, as partners can only estimate the force they reproduce through the finger pad of one finger. For a fair comparison between individuals and pairs, individuals should only be able to estimate the force they reproduce through the finger pad of one finger alone. Walsh *et al*.^15^ and we tested the accuracy of individuals in reproducing forces in two conditions, one where an individual reproduced the sensed force back onto the sensing finger using their contralateral finger, and another where only one finger is used to sense and reproduce the force (see supplementary materials). Walsh *et al*.’s and our results demonstrate that individuals over-reproduce small and under-reproduce large forces with one finger, so pairs should converge in force, and will not escalate indefinitely.

Importantly, our study neither advocates nor refutes the mechanism of predictive sensory attenuation^16^, but questions whether such neural processing of the force percept alone can explain the behaviour of pairs exchanging forces. Both separated and face-to-face pairs should have been affected by the bias caused by neural processing equally, hence no difference should be observed between these two groups if it is exclusively responsible. However, our results show that separated and face-to-face pairs behave differently from one another, implying that neural processing alone cannot explain sensorimotor exchanges in force between pairs.

What caused face-to-face pairs to behave differently from separated pairs and individuals? Studies^1^,^5^ have reported significant deviation in a subject’s behaviour under the gaze of other humans, and that people maintain a larger distance when approaching an avatar from the front rather than the back^8^. Similarly, our face-to-face pairs may have reproduced the force differently due to the other’s gaze, which was not present in separated pairs. Another possibility is that face-to-face pairs (who were strangers to one another) were physically too close and encroached each other’s personal spaces^2^,^17^. Both separated and face-to-face pairs were physically equidistant from each other, but the curtain may have established a boundary such that separated partners felt that their personal space was not under threat. Furthemore, subjects might have used visual and/or tactile information of the partner’s reaction to modulate the force reproduced onto the partner, or have extracted visual information about the force reproduced onto the partner that could have biased their accuracy in reproducing the sensed force. The strong coupling between the influenced face-to-face partners suggests such an interactive component whereby one partner is biased into under-reproducing the force due to the partner who is doing so. A further study is necessary to test which factors are the most influential during exchanges in force, and also to identify whether the perception of the partner’s force per se was affected, or whether partners under-reproduced an accurately perceived partner’s force. The strength of the coupling could also be tested by inducing one partner in a separated pair to under-reproduce the force to observe whether the other partner under-reproduces the force as well.

The exact social factors that influence face-to-face exchanges of force are unknown, but our results show that studies on sensorimotor interaction between humans^18^,^19^,^20^ should take such social influences into account. Otherwise, the difference in a partner’s behaviour may be wrongly attributed to an interactive strategy rather than the influence of social factors. To avoid such confounds, we suggest the use of a curtain to mitigate the effect of social factors when investigating motor adaptation during sensorimotor interaction. The ecological validity of our study is limited, as exchanges of force between humans in real-life would occur in close proximity than our study, and without any rest-period between exchanges. However, our controlled experimental paradigm has revealed that partners should not face each other during interaction if only motor adaptation is to be investigated.

## Methods

Experiments were performed at the Department of Bioengineering at Imperial College London. The subjects gave informed consent for their participation in the experiments which were conducted according to the principles in the Declaration of Helsinki and approved by the Imperial College Research Ethics Committee. The Edinburgh inventory was used to determine the handedness of our subjects in the individual, separated and face-to-face conditions (mean ± standard error of 0.78 ± 0.03, 0.86 ± 0.04 and 0.84 ± 0.05 respectively). The mean age of participants in each condition was 27.5 ± 3.7 for individuals, 26.3 ± 3.2 for separated and 27.8 ± 2.9 years old for the face-to-face groups.

### Experimental procedure

Each robotic device was equipped with a motor and a torque sensor^21^. During individual force estimation, the initial force was selected randomly from a uniform distribution between 0.5–10N. Once the initial force was selected, the robot linearly ramped up to this force in 1 second, maintained it for 5 seconds then ramped back down in 1 second. After a rest period of 5 seconds, subjects were prompted to reproduce the force back onto the lever, which was locked in place using position control. In individual force estimation, this process of sensing and reproducing the force was repeated 30 times. For pairs, the robot applied an initial force of 2, 4, 6, 8 or 10N to a partner who was randomly selected; after a rest period of 5 seconds, this partner reproduced a force that was sent to the other partner’s finger through the robotic levers, which were rigidly connected through software. Pairs exchanged this force 6 times, which formed one trial. Pairs experienced 15 trials in total, 3 trials per force level. When the minimum force threshold of 0.5N was crossed, the reproducing partner reproduced a constant force for 5 seconds. The force in the last 3 seconds was used for the analysis as subjects took time to climb and stabilise. One outlier face-to-face pair was removed from the analysis.

### Force reproduction

Data from the individual force reproduction were fit with a linear mixed-effects model with reproduced force *F* as the dependent variable and the perceived force, *F*_*p*_, as the predictor in the form


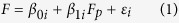


where *β*_0*i*_ is the fixed intercept, *β*_1*i*_ the slope and *ε*_*i*_ is the unexplained variance of the reproduced force for each subject *i*, which is our random factor. We compared the accuracy of separated and face-to-face pairs in reproducing a force by adding the group factor *I* in the model,


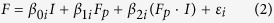


which was assumed to affect both the slope and the intercept of the force reproduction curve.

Identifying subpopulations in face-to-face pairs. In both separated and face-to-face pairs, the mean reproduced force from each subject was plotted as a function of the mean force from the partner. The Shapiro-Wilk test revealed that these data from separated pairs were normally distributed (see Fig. 2C), but they were not in face-to-face pairs (Fig. 2F). We used a Gaussian mixture model to cluster the face-to-face data; increasing the number of clusters to minimise Akaike Information Criterion was found to break up the “influenced cluster” (bottom-left subjects in Fig. 2F) into smaller ones. Thus, we concluded that two subpopulations represent our face-to-face data: one subpopulation whose mean exchanged force was similar to separated partners, and another subpopulation that was different from them, as judged by a one way analysis of variance of the mean exchanged force with subpopulation/group as a factor.

## Additional Information

**How to cite this article**: Takagi, A. *et al*. Facing the partner influences exchanges in force. *Sci. Rep*. **6**, 35397; doi: 10.1038/srep35397 (2016).

## Supplementary Material

Supplementary Information
