# Supplementary material for: Facing the partner influences exchanges in force
Source: Sci Rep. 2016 Oct 14;6:35397. doi: 10.1038/srep35397 (PMC5064314; doi:10.1038/srep35397)
Supplement: Supplementary Information [file srep35397-s1.doc]

**Supplementary Materials**

**Facing the partner influences exchanges in force**

Atsushi Takagi*^†^, Carlo Bagnato* and Etienne Burdet

Department of Bioengineering, Imperial College London, South Kensington Campus, London SW7 2AZ, UK.

*equally contributing authors, ^†^[atsushi.takagi07@imperial.ac.uk](mailto:atsushi.takagi07@imperial.ac.uk)

**Individual force reproduction**

We tested the same 8 subjects in two different conditions to test an individual’s ability to sense and reproduce a force (see Fig. S1A). In *push-back* reproduction, the subject passively sensed a force from a robot (which sent forces picked from a uniform distribution between 0.5-10N) and then reproduced the force by pushing back onto the lever using the same finger. In *squeezing reproduction*, the force was sensed passively by the right finger, and then reproduced onto this finger by pressing a connected lever using the contralateral finger. Fig. S1B shows the reproduced force as a function of the force from the robot in both conditions, revealing that *push-back* is less accurate than *squeezing* reproduction.

Why is *push-back* reproduction less accurate with significantly larger inter-subject variability than *squeezing*? Critically, during *squeezing* reproduction, the sensing and reproduction of force involves the same sides of a finger. However, in *push-back* reproduction the sensing finger is squeezed passively between two surfaces, but the reproduced force can only be estimated by one surface of the finger pad. The subject must match the sensation between a two-sided compression of the finger with a pressure on the finger pad, which is likely more difficult than the *squeezing* condition. Indeed, out of 8 subjects in Walsh’s study, “2 subjects were unable to perform the task” as “they found the task too difficult”. This difficulty in matching two distinctly different sensations may be the reason why inter-subject variability in the *push-back* reproduction is so large.

Although our results demonstrate the same relative relationship of the *squeezing* reproduction being more accurate than the *push-back* reproduction as Walsh’s study does, the accuracy of both the *squeezing* and *push-back* conditions are slightly different between the two studies. Such a difference may occur as force is sensed by mechanoreceptors as pressure. Thus, the size and shape of the contact between the finger and the force-producing lever will change the accuracy in reproducing a force.


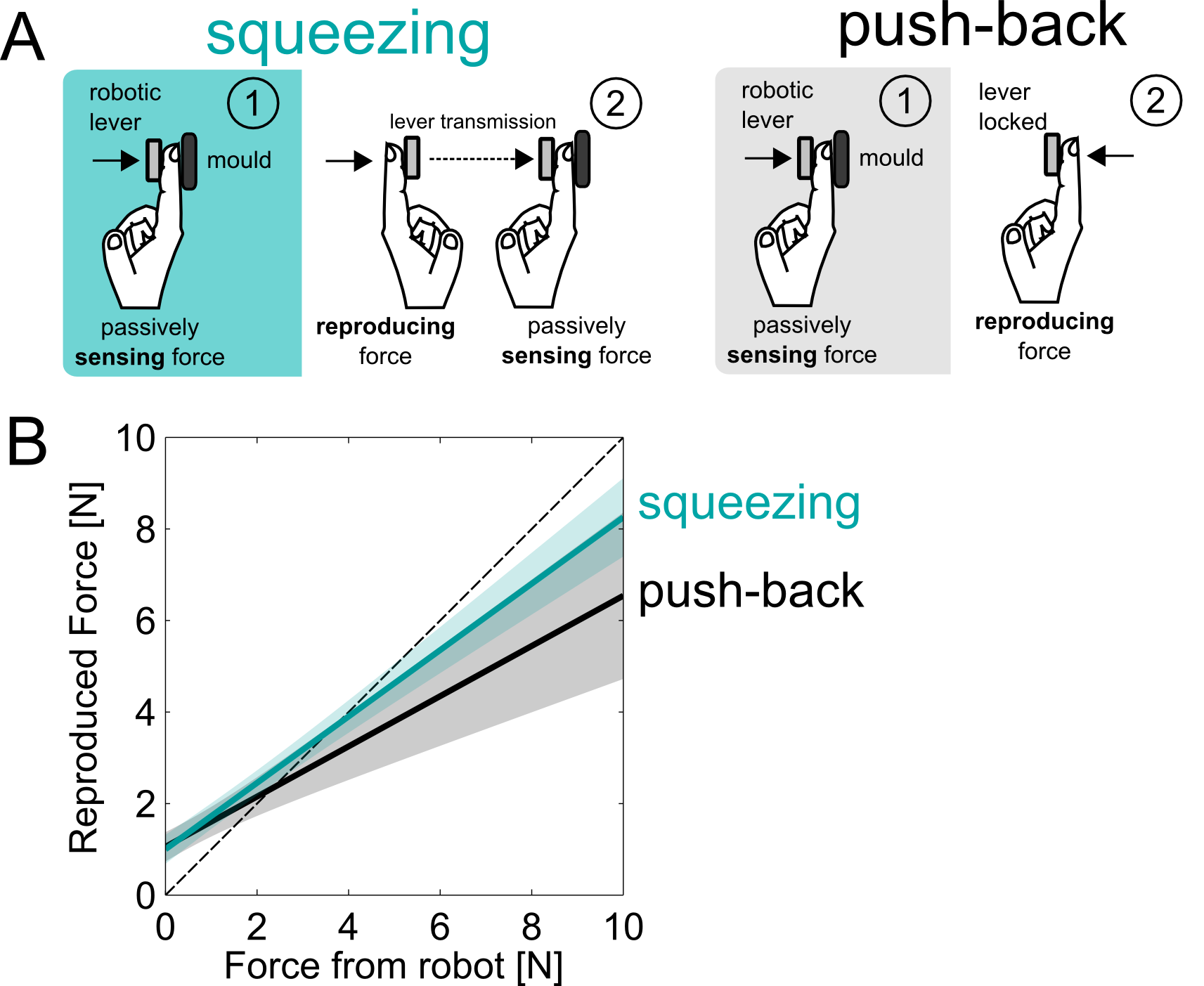


**Figure S1 | Accuracy of reproduced force depends on sensing and reproduction conditions.** (**A**) Schematic showing the *push-back* and *squeezing* reproduction conditions. In *push-back reproduction*, the force is sensed passively on the right index finger, and is reproduced on the locked lever using the same finger. In *squeezing reproduction*, the right index passively senses the force, which is reproduced by the left index onto the right index through a rigidly connected lever system (enabled by software). (**B**) Reproduced force as a function of the force from the robot for *push-back* and *squeezing* reproduction conditions. *Squeezing* was more accurate than *push-back*, which was also significantly more variable between-subjects.

**References**

1. Walsh, L. D., Taylor, J. L. & Gandevia, S. C. Overestimation of force during matching of externally generated forces*. J. Physio*l**. 58**9, 547–557 (2011).
